# Ambulatory care sensitive hospitalizations after implementation of the master plan in Minas Gerais

**DOI:** 10.11606/S1518-8787.2018052017330

**Published:** 2018-07-13

**Authors:** Luciano José Arantes, Helena Eri Shimizu, Edgar Merchán-Hamann

**Affiliations:** I Universidade de Brasília. Programa de Pós-Graduação em Ciências da Saúde. Brasília, DF, Brasil; II Universidade de Brasília. Faculdade de Ciências da Saúde. Departamento de Saúde Coletiva. Brasília, DF, Brasil

**Keywords:** Primary Health Care, Ambulatory Care, Hospitalization, Health Care Quality, Access, and Evaluation, Ecological Studies, Atenção Primária à Saúde, Assistência Ambulatorial, Hospitalização, Qualidade, Acesso e Avaliação da Assistência à Saúde, Estudos Ecológicos

## Abstract

**OBJECTIVE:**

To describe the rate of ambulatory care sensitive hospitalizations in groups of cities according to population size and to analyze its association with the coverage of the Family Health Strategy after the implementation of the Master Plan for Primary Health Care in Minas Gerais, Brazil.

**METHODS:**

This is an ecological study with 452 cities grouped according to population size, with data from 2004 to 2007 and 2010 to 2013. We used the Kolmogorov-Smirnov test to verify the distribution of the data in the groups. We used the Wilcoxon test for paired data or the paired Student’s t-test to compare the rate of ambulatory care sensitive hospitalizations before and after the Master Plan for Primary Health Care. We used the simple linear regression test to analyze the association between variables. We performed statistical analyses using the Statistical Package for the Social Sciences, with a significance level of 5%.

**RESULTS:**

The rate of ambulatory care sensitive hospitalizations decreased significantly after the Master Plan for Primary Health Care in the large and mid-sized groups (p < 0.05). There were positive correlations between coverage with Family Health Strategy and the rate of ambulatory care sensitive hospitalizations in the mid-sized and large groups (p < 0.05).

**CONCLUSIONS:**

Actions were carried out to implement the Master Plan for Primary Health Care. However, more investments are needed to improve the effectiveness of the Primary Health Care, with permanent confrontation of complex issues that affect the quality of services, which can lead to a significant reduction of the rates of ambulatory care sensitive hospitalizations.

## RESUMO

**OBJETIVO:**

Descrever a taxa de internação por condição sensível à atenção primária à saúde em grupos de municípios conforme o porte populacional e analisar sua associação com a cobertura pela Estratégia Saúde da Família após a implantação do Plano Diretor da Atenção Primária à Saúde em Minas Gerais.

**MÉTODOS:**

Estudo ecológico com agrupamento de 452 municípios segundo o porte populacional, comparando dados de 2004 a 2007 e 2010 a 2013. O teste de *Kolmogorov Smirnov* foi utilizado para verificar a distribuição dos dados nos grupos. O teste de *Wilcoxon* para dados pareados ou teste t Student pareado foi usado para comparação da taxa de internação por condição sensível à atenção primária à saúde antes e após o Plano Diretor da Atenção Primária à Saúde. O teste de regressão linear simples foi utilizado para analisar a associação entre as variáveis. Foram realizadas análises estatísticas pelo *Statistical Package for the Social Sciences*, com nível de significância de 5%.

**RESULTADOS:**

A taxa de internação por condição sensível à atenção primária à saúde diminuiu significativamente após o Plano Diretor da Atenção Primária à Saúde no grupo com grande e médio porte populacional (p < 0,05). Houve correlações positivas entre a cobertura com Estratégia Saúde da Família e a taxa de internação por condição sensível à atenção primária à saúde nos grupos com porte populacional médio e grande (p < 0,05).

**CONCLUSÕES:**

Foram empreendidos esforços com a implantação do Plano Diretor da Atenção Primária à Saúde. Entretanto, é preciso que mais investimentos sejam realizados para contribuir para a melhoria da efetividade da Atenção Primária à Saúde, com enfrentamento permanente de questões complexas que incidem sobre a qualidade dos serviços, gerando redução significativa das taxas de internação por condição sensível à atenção primária à saúde.

## INTRODUCTION

Primary Health Care (PHC) is the first level of contact between persons and the health system. It involves carrying out actions for the promotion and protection of health, prevention of diseases, diagnosis, treatment, rehabilitation, harm reduction, and maintenance of health, which can reduce hospital admissions and emergency care^1^ ^,^ ^2^.

The Family Health Strategy (FHS) was adopted in Brazil as a priority for the expansion of these services since 1994. States and cities have developed proposals in line with national regulations to improve services at this level of care. Among them, we can mention the Master Plan for Primary Health Care (PDAPS), which was an initiative of the State Department of Health of Minas Gerais (SESMG), implemented from 2006 to 2010 in the state. Its purpose was to improve the quality of health services in cities with the standardization of the PHC work and actions for clinical management^3^.

The summary objective of the PDAPS, defined by SESMG, was the decrease in ambulatory care sensitive hospitalizations (ACSH). This objective comes from the ability of the PHC in avoiding or decreasing the occurrence of hospital admissions for a specific group of causes, which would contribute with decreased hospital expenses^3^.

The PDAPS was implemented through educational workshops planned to take place in the 853 cities of the state in three phases: from 2006 to 2007, from 2008 to 2009, and from 2009 to 2010, covering 28, 455, and 370 cities, respectively^3^.

The contents covered in the workshops were related to the construction of health care networks, analysis of PHC in the city, local diagnosis, local/city programming, reception and risk classification, family approach, management contract, laboratory diagnostic support system, family health records, monitoring, and evaluation^3^.

The methodology used in the educational workshops was based on the identification of problems, stimulated reflection of practices, and elaboration of contextualized solutions. It was considered the largest training plan in PHC in the state, with more than 50,000 professionals attending it^3^ ^,^ ^4^.

Studies on PDAPS point out contributions to the organization of the PHC, multi-professional integration, and standardization of actions according to guidelines, in the reception process, in the mapping of areas of action of PHC teams, in local programming, in the development of clinical protocols and guidelines to normalize processes, actions, and behaviors in PHC, in the reduction of infant mortality, and in the construction of health networks^5–8^.

Despite the positive aspects of the PDAPS, few studies have been published based on city health indicators after the educational workshops. There is also a gap in the knowledge of whether or not ACSH have declined after implementation, during a period in which the federal and state governments encouraged the expansion of FHS coverage in the cities of Minas Gerais.

The objective of this study is to describe the rate of ACSH in groups of cities according to population size and to analyze its association with the coverage of the Family Health Strategy after the implementation of the Master Plan for Primary Health Care in Minas Gerais, Brazil.

## METHODS

As the implementation of the PDAPS did not occur simultaneously, we chose to carry out an ecological study with the group of cities that participated in phase 2, in 2008 and 2009, because of the greater number of cities involved. The cities were part of the area covered by the Regional Health Units (RHU) of Barbacena, Diamantina, Divinópolis, Ituiutaba, Januária, Juiz de Fora, Leopoldina, Manhumirim, Montes Claros, Patos de Minas, Pedra Azul, Pirapora, Ponte Nova, São João Del Rei, Teófilo Otoni, Ubá, Uberlândia, and Unaí.

We included cities that exclusively participated in phase 2 and all educational workshops proposed in 2008 and 2009. Two cities of the RHU area of Ituiutaba were excluded, as they only participated in the initial workshops of phase 2, and one city of the RHU of Juiz de For a was also excluded, as it participated in phases 2 and 3. Thus, the study sample consisted of 452 cities in the state.

The cities participating in phase 2 of the PDAPS were grouped according to population size, according to data from the Brazilian Institute of Geography and Statistics (IBGE): < 49,999 inhabitants (small), between 50,000 and 99,999 inhabitants (mid-sized), and > 100,000 inhabitants (large). The analysis groups consisted of 427 small cities, 17 mid-sized cities, and eight large cities.

To calculate ACSH, our reference was the list used at the time of implementation of the PDAPS, described in State Resolution 1,093, of December 29, 2006, which includes problems related to mental health. The ACSH were classified according to the diagnostic group and the International Classification of Diseases, 10th revision (ICD-10).

We counted the ACSH of persons living in Minas Gerais who received care in the state itself or in those close to the territorial border: Bahia, Goiás, Espírito Santo, Rio de Janeiro, São Paulo, and Federal District. Thus, we considered the possible regional movement of users to receive care.

We calculated the annual rate of ACSH by dividing the number of ACSH by the population estimated by the IBGE in the year evaluated, with the exception of 2010, for which we used data from the demographic census. The result of this division was multiplied by 10,000. The data were collected from the website of the Information Technology Department of the Brazilian Unified Health System (DATASUS). The numerator data came from the Hospital Information System of the Brazilian Unified Health System (SIH-SUS) and the denominator data came from IBGE. The records with hospitalization data were processed in the program Tabwin version 3.5 and inserted in Excel^®^ spreadsheets to calculate the indicator. We calculated the mean and median rates of ACSH for each group annually. We adopted the median as the most reliable parameter for analysis, because of the influence of extreme values in the calculation of the mean.

For statistical analysis, we calculated the mean rate of ACSH in each city from 2004 to 2007, and from 2010 to 2013, and we obtained a mean rate before and after the PDAPS. This allowed us to compare two different moments of the intervention. For each group, we obtained a paired data set formed by the rate of ACSH of each city before and after the PDAPS.

We verified whether the data set (mean rates of ACSH) in each group analyzed had a normal distribution, both for 2004 to 2007 and 2010 to 2013, using the Kolmogorov-Smirnov test, with significance of 5%. This test was chosen because there was no assumption regarding the distribution of the data in the groups studied. In addition, we could analyze the distribution of data in the group with a small number of cities, such as the group of large cities (n = 8).

To compare the data set (mean rates of ACSH) before and after implementation of the PDAPS, we applied the paired Student’s t-test in the groups formed by the cities of Minas Gerais (n = 853), participating in the PDAPS (n = 452), and with a small population (n = 427). This procedure was used because the data presented normal distribution (p > 0.05) in these groups. We did not find a normal distribution of the data in the groups formed by mid-sized (n = 17) and large cities (n = 8). We used the non-parametric Wilcoxon test for paired data to compare the rates of ACSH before and after the implementation of PDAPS. This test is used when the requirements for the paired Student’s t-test are not met and it is best suited for small numbers. These analyses, with significance level of 5%, showed whether the rate of ACSH increased or decreased after the implementation of PDAPS in each group analyzed.

We applied the simple linear regression test to evaluate the correlation between FHS coverage (independent variable) and rate of ACSH (dependent variable) from 2004 to 2007 and from 2010 to 2013. We collected data on monthly population covered by the FHS from the website of the Department of Primary Care of the Ministry of Health. Thus, we calculated the mean monthly values to obtain the annual coverage value of each city. The null hypothesis was that the independent variable was not correlated with the dependent variable. The alternative hypothesis was that the groups with high percentage of FHS coverage presented small values in the rate of ACSH (negative correlation).

The statistical analyses were performed in the program Statistical Package for the Social Sciences (SPSS), version 22. The study was not submitted to the Ethics Committee because it uses secondary data, available online.

## RESULTS

**Figure 1:**
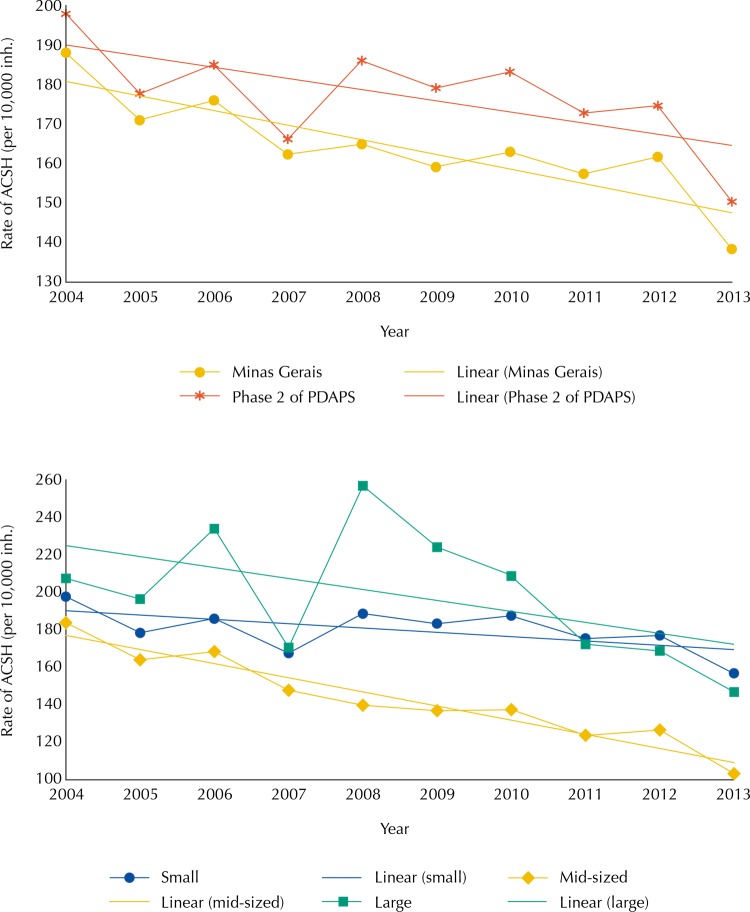
Median rate of ACSH (per 10,000 inh.) in State of Minas Gerais, Brazil, and group of cities participating in phase 2 of the PDAPS according to population size, 2004 to 2013.

ACSH: Ambulatory Care Sensitive Hospitalizations; PDAPS: Master Plan for Primary Health Care; inh.: inhabitants

The median rate of ACSH was higher in the group of cities that participated in phase 2 of the PDAPS than in the State of Minas Gerais. Both showed a decrease over the analyzed period (Table 1 and Figure 1).

**Table 1:** Mean and median rate of ACSH (per 10,000 inhabitants) in State of Minas Gerais, Brazil, and group of cities participating in phase 2 of the PDAPS according to population size, 2004 to 2013.

Ecological units	Indicator	Year									

		2004	2005	2006	2007	2008	2009	2010	2011	2012	2013
Minas Gerais	Mean	205.58	187.74	191.52	175.54	187.55	177.01	189.90	184.02	187.75	166.29
	Median	188.01	171.03	175.96	162.35	164.92	159.10	162.95	157.36	161.68	138.34
Phase 2 of PDAPS	Mean	214.89	193.14	201.67	182.93	203.95	190.92	208.47	197.34	198.79	170.89
	Median	197.92	177.73	185.02	166.20	186.08	179.14	183.25	172.80	174.65	150.33
Population size											
Small	Mean	216.06	193.74	202.26	184.24	204.65	192.38	211.12	200.34	202.03	173.79
	Median	197.74	178.41	185.99	167.51	188.70	183.19	187.46	175.31	176.91	156.59
Mid-sized	Mean	184.18	171.29	164.77	147.57	155.11	143.92	142.72	136.47	137.53	116.63
	Median	183.76	164.00	168.37	147.74	139.75	136.85	137.37	123.68	126.55	103.22
Large	Mean	217.73	207.58	248.81	188.09	270.38	213.17	206.65	166.66	156.10	131.35
	Median	207.39	196.38	233.91	170.55	256.80	224.05	208.61	172.28	168.74	146.82

ACSH: Ambulatory Care Sensitive Hospitalizations; PDAPS: Master Plan for Primary Health Care

The medians of the rates in the groups according to population size varied between 183.8 and 207.4 at the beginning of the period (2004), and between 103.2 and 156.6/10,000 inhabitants at the end (2013) (Table 1).

Considering the cities participating in phase 2 of the PDAPS, the group formed by large cities showed the highest rates of ACSH. The group of small cities had lower rates than the group of large cities in most of the evaluated years, although they were higher after the PDAPS in the last three years of the period (2011 to 2013). The lowest rates were identified in the mid-sized group (Figure 1). The evaluated groups showed a decrease in the median rates of ACSH (Figure 1).

The rate of ACSH decreased after the implementation of the PDAPS in most of the cities analyzed, with statistical significance in most groups (p < 0.05) (Table 2). There was no statistical difference after PDAPS in the group of small cities (p > 0.05) (Table 2).

**Table 2:** Comparison of the rate of ACSH (per 10,000 inhabitants) in State of Minas Gerais, Brazil, and group of cities participating in phase 2 of the PDAPS according to population size, 2004 to 2007 and 2010 to 2013.

Ecological units	n	Number of cities		Mean rate of ACSH		p
	
		Increase in the rate of ACSH	Decrease in the rate of ACSH	2004 to 2007	2010 to 2013	
Minas Gerais	853	329	524	190.10	181.99	0.000^a^
Phase 2 of PDAPS	452	185	267	198.16	193.87	0.009^a^
Population size						
Small	427	181	246	199.08	196.82	0.066
Mid-sized	17	3	14	166.95	133.34	0.001^a,b^
Large	8	1	7	215.55	165.19	0.014^a,b^

ACSH: Ambulatory Care Sensitive Hospitalizations; PDAPS: Master Plan for Primary Health Care

^a^ Significant results (p < 0.05).

^b^ Results from the paired t-test; the other p values, from the Wilcoxon test for paired data.

**Figure 2:**
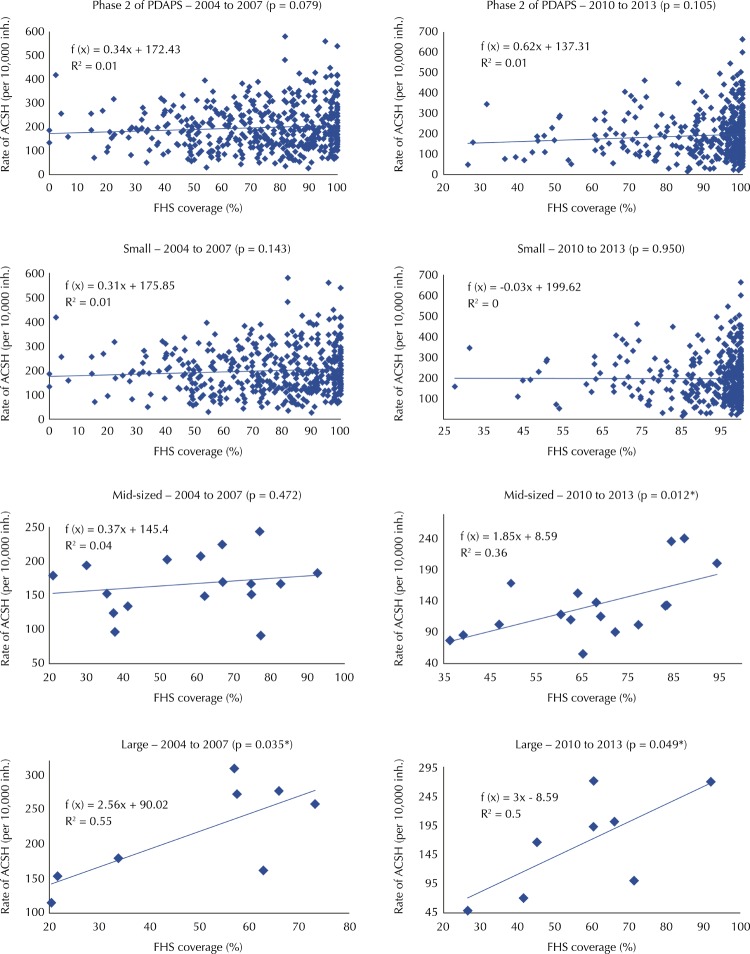
Scatter diagrams of FHS coverage and rate of ACSH (per 10,000 inh.) in cities participating in phase 2 of the PDAPS according to population size, State of Minas Gerais, Brazil, 2004 to 2007 and 2010 to 2013.

ACSH: Ambulatory Care Sensitive Hospitalizations; PDAPS: Master Plan for Primary Health Care; FHS: Family Health Strategy; inh.: inhabitants

The correlation between FHS coverage and rate of ACSH was statistically significant and positive in the group of large cities before and after PDAPS, and in the group of mid-sized cities after implementation (p < 0.05) (Figure 2). On the other hand, this association was weak in the group of mid-sized cities (R = 0.596 from 2010 to 2013). The correlation between the variables was moderate before and after the implementation of PDAPS in the group of large cities (R = 0.742 from 2004 to 2007 and R = 0.709 from 2010 to 2013). Although this group had a small size (n = 8), the regression line could be used to represent the points obtained with the analysis because the regression test presented a statistically significant result (p < 0.05).

* Significant result (p < 0.05).

Table 3 presents the summary of the results of the points addressed in the study.

**Table 3:** Summary of the results. State of Minas Gerais, Brazil, and group of cities participating in phase 2 of the PDAPS according to population size, 2004 to 2007 and 2010 to 2013.

Ecological units	Rate of ACSH		Association between FHS Coverage and rate of ACSH	
	
	Trend 2004 to 2013	Comparison 2004 to 2007 x 2010 to 2013	2004 to 2007	2010 to 2013
Minas Gerais	Decreasing	Decrease*	-	-
Phase 2 of PDAPS	Decreasing	Decrease*	-	-
Population size				
Small	Decreasing	Decrease	-	-
Mid-sized	Decreasing	Decrease*	-	Positive and weak*
Large	Decreasing	Decrease*	Positive and moderate*	Positive and moderate*

ACSH: Ambulatory Care Sensitive Hospitalizations; PDAPS: Master Plan for Primary Health Care; FHS: Family Health Strategy

* Significant results (p < 0.05).

## DISCUSSION

The decrease in ACSH from 2004 to 2013 followed the trend identified before this research by other authors in State of Minas Gerais. This is probably linked to the de-hospitalization of patients with mental disorders and the increase of vaccination coverage for children^9^ ^,^ ^10^.

The annual rates of ACSH, higher in the group of cities participating in phase 2 of the PDAPS than in the State of Minas Gerais, may be related to the fact that the sample covers cities with greater need of social investment in the state, such as those located in the North and Northeast. Therefore, they indicate the need to continue investing in health actions to reduce the values found.

The rates of ACSH were higher in the large and small groups, but they were higher in the small group in the last three years. This fact points to the need to implement measures that seek to decrease ACSH in these groups, such as those aimed at improving the quality of FHS/PHC and health and socioeconomic conditions. Population aging should also be considered, since the literature mentions that the main causes of hospitalization in older populations are for ACS conditions^11^.

Low education level, low income, and age over 60 years increase the chance of ACSH^11^ ^,^ ^12^. With a low educational level, users may have less knowledge on the health care and difficulty understanding the proposed therapy. This can lead to hospitalizations when the situation of the disease deteriorates. In addition, low education level may lead to less employment opportunities and may adversely affect health care in these more vulnerable groups. In addition to affecting the access of persons to health services and their pattern of use, low income affects the access to drugs, particularly those that must be purchased, as well as other necessary inputs for health maintenance^13^.

The main causes of hospitalization in older adults have been for ACS conditions, including a seven-fold higher risk of hospitalization for these conditions^11^ ^,^ ^14^. This is because older adults are subject to greater physical vulnerability, difficulty walking, less interaction with persons, and financial problems, besides the low use of preventive actions of PHC and little understanding about primary health care.

A higher number of older adults are hospitalized for ACS conditions in locations with an older population, which generates an increase in health system expenditures^15^. For older adults with low income and education level, the situation is exacerbated and can cause more frequent hospitalizations^16^.

Faced with the possibility of influencing these determinants in ACSH, governmental authorities should act on the improvement of the socioeconomic conditions of the population. Such actions can contribute more to the health status of populations when compared to medical interventions^17^ ^,^ ^18^.

The abrupt increase in the median rate of ACSH in 2008, identified in the large group, was related to the increase in hospitalizations for mental health and bacterial pneumonia (Figure 1). This fact shows the low case-resolving capacity of the PHC for these pathologies. Organizational factors related to the work process of the FHS can also impair the access of users to PHC services and lead to an increase in pathologies that, because of a lack of diagnosis and effective control, can be exacerbated and solved only at the hospital level^19^. This issue undermines the credibility of the FHS/PHC as the focal point of care and, as a consequence, makes users choose hospitals as their first option. Therefore, ACSH can be high, especially in large cities where users have more access to these establishments^19^.

The rates of ACSH are influenced not only by issues related to PHC but also to secondary and tertiary care. The resolving of cases in PHC and in specialized care, the policies adopted in the study period for the expansion or release of beds, and the surgeries performed in outpatient clinics may have influenced the results found.

Large cities, in addition to the issue of accessibility to the service, generally have more beds for hospitalization than small and mid-sized cities. This fact may induce the physician to hospitalize more, especially when working in poorer areas, such as outskirts of large centers, considered as priority sites for the implementation of FHS^19^. The lower rates of ACSH in the mid-sized group indicate that PHC is more effective in this group, even though it has more difficult expanding its PHC because of the conflict in prioritizing it in view of the demands for the implementation of services of medium and high complexity.

The mid-sized and large groups seem to have benefited from PDAPS because of the significant reductions in the rates of ACSH. This was not evidenced in the group of small cities, which suggests that the activities carried out with the implementation of PDAPS were far from the working conditions and peculiarities of some places, as reported by some authors^20^.

We expected that the expansion of the FHS would induce the decrease in the rate of ACSH, mainly because of the work process adopted, which broadens the instruments needed for the case-resolving ability of health actions^21^. The lack or positive correlation identified between the variables, even if weak or moderate, shows flaws in the quality of the FHS, and it points to the need to investigate possible factors associated with FHS that lead to this type of result.

The decrease of ACSH may be related to the performance of the FHS in some situations, but this relation is not confirmed as a constant in all evaluations^22^. This is due to the possible variation in the quality and intensity of FHS activities from one place to another^23^. In addition, variations can be attributed to the characteristics of the cities, each health region, and prevalent pathologies^22^ ^,^ ^24^ ^,^ ^25^.

The increase in population covered by the FHS is considered as an explanatory factor for the reduced rate of ACSH, including in Minas Gerais^26^. However, national studies have identified associations similar to those found in this research in Acre, Amazonas, Pará, Espírito Santo, and in the city of Juiz de Fora, State of Minas Gerais^13^ ^,^ ^16^ ^,^ ^27^. A research that has covered the regional health services of Minas Gerais of 2000 and 2010 has also found similar correlations, and it has identified a negative association between FHS coverage and rate of ACSH only in one regional unit^25^.

The increase in the rates of diagnosis for ACSH may be a result of improved access provided by the expansion of PHC services. This fact allows the diagnosis of cases that will affect hospitalization indicators^28^. Such situation can occur in areas that, historically, have had limited access to health services, temporarily increasing hospitalizations with the implementation of FHS^29^. This possibility seems to have happened in the groups with a positive correlation between coverage and rate of ACSH, as we identified an increase in FHS coverage after the implementation of the PDAPS, especially in the stratum of large population size, which presented a moderate correlation between the variables both before and after implementation. The positive correlation indicates the need for continuous investments in order to expand the provision of PHC/FHS qualified services and actions. It is hoped that the repressed demands for hospitalizations will decrease and the FHS will play its intended role in the decrease of ACSH, by considering the demands of each city and health regions. There are other factors not tested in this study that may be associated with ACSH, in addition to FHS coverage. Therefore, it is important to verify in other research studies whether the associations found are kept in the presence of other elements. For this end, multiple analysis models should be used.

This research has limitations. Even though the cities of each group have common population size, the results for each group cannot be extrapolated to the city level, as the analyses were performed considering the groups of cities. Inaccuracies may also occur in the record of hospitalizations in the SIH-SUS^16^ ^,^ ^28^ ^,^ ^30^. However, the online availability of the data on the DATASUS website can increase their use for epidemiological purposes, which contributes to their criticism and improves the data quality^2^ ^2^ ^,^ ^30^.

This study identified positive results for the rate of ACSH after the implementation of the PDAPS in the study population, particularly in the mid-sized and large groups. However, the PHC still faces obstacles for its consolidation in local health systems, as the increase in coverage with FHS teams is not followed by a significant decrease in the rates of ACSH. More investments need to be made to improve the effectiveness of the PHC, with ongoing confrontation of complex issues that affect the quality of health services and actions, leading to a significant decrease in the rates of ACSH.
